# On the Question of the Innocuity in Certain Physiological Secretions in Syphilis

**Published:** 1878-02

**Authors:** James Nevins Hyde

**Affiliations:** Chicago


### ON THE QUESTION OF THE INNOCUITY OF CERTAIN PHYSIOLOGICAL SECRETIONS IN SYPHILIS.

By James Nevins Hyde, M. D., Chicago.

1. *Ist Syphilis durch die Milch Uebertragbar?* [Is Syphilis transmissible through the milk?) R. Voss. (St. Petersburg. Med. Wochenschrift, No. 23, 1876.]
2. *Recherches sur la Question de l'innocuite du Lait provenant de Nourrices Syphilitiques.* [Examination of the question respecting the innocuity of the milk of syphilitic nurses.] Ernest Gallois, Paris, 1877.
3. *Sulla non Trasmissibilita della Sifilide per mezzo del Latte.* [On the non-transmissibility of syphilis by the medium of milk.] Tommaso De Amicis. (Annal. Clin. dello Osped. Incurab. An. II., Sept. and Oct., 1877, F. 5, p. 278.)
4. *Two Cases of Transmission of Syphilis Through the Male Element of Reproduction.* M. H. Jordan. (Am. Jour. of Obstet., Jan'y, 1878, p. 126.)
5. *Recherches sur la Non-inoculabilite Syphilitique du Sperme.* [Investigation of the question of the non-inoculability of the semen in syphilis.] H. Mireur. (Ann. de Dermatol et de Syphilig., T. 8, No. 6, p. 423.)
6. *Syphilis Communicated by Tattooing.* F. F. Maury and C. W. Dulles (Amer. Jour. of the Med. Sci., Jan'y, 1878, p. 44.)

Syphilis is unquestionably a disease of surprises. There is no organ nor tissue of the human body which it may not invade; and it would be indeed strange, if, with such an extended field of attack, its incursions should not be at times unexpected, both as to date, direction, and severity. The recognized predilection for special tissues, of many common pathological processes, often furnishes a clue to their probable course and issue, but this is not true of many of the complications in syphilis. It is rare, also, to note in non-specific disorders that complete inversion of the established sequence of events, by which phenomena of usually tardy occurrence not only become precocious, but are even succeeded by symptoms ordinarily present only in the incipient stage of the disease. This is, however, not rarely observed in syphilis.

The result is two-fold. There are observers whose maxim seems to be, "once a syphilitic, always a syphilitic." They ignore the positive proofs afforded by reinfection, from which it is possible to conclude that syphilis is eradicable; and, if an individual but admits that he has once suffered from chancre, they hasten to refer to it every subsequent disorder of the cutaneous, osseous, muscular, and nervous systems. Others err in a different direction. They are unwilling to believe that an insignificant lesion, which possibly existed a decade or more of years ago, can explain the obscure phenomena with which they are confronted to-day. Even so late as the year 1873, Després, for example, took occasion to announce in public, his disbelief in the existence of gummy tumors as specific products.

Of all the diseases in our nomenclature, there is probably none with regard to which so many points have been hotly contested, and where so many great names have been found on either side of the controversy.

One of these disputed questions, and by no means a new one, respects the possibility of transmitting syphilis through the medium of physiological secretions. Can it be shown that the tears, sweat, saliva, mucus and semen of a syphilitic individual, when not commingled with blood or pathological products, are incapable of producing the disease in a healthy person, whether these secretions be swallowed or introduced hypodermically? It is hardly necessary to observe that the contagious qualities of the blood in syphilis have never been questioned since Prof. Pellizari, of Florence, employed it for the transmission of the disease to his pupil, Bargioni, an experiment the results of which are embodied in almost every treatise on the disease.

With respect to the transmissibility of syphilis in milk, we find the question under discussion as early as the latter part of the 15th century, and continuing *sub judice* up to this day—a discussion in which Hunter, Swediaur and Cullerier, with others, have held that it was innocuous, while such authors as Astruc, Bell and Diday have advanced a contrary opinion. Gallois (2), in an interesting sketch of the history of this controversy, mentions the following names of authors who have contributed to it during tho last fifteen years: Melchior Robert (1861), Langlebert (1864), Ricordi and Plaite (1865), and Cerasi (1866), all contagionists; Rollet (1861, 1866 and 1875),

Pellizari (1866), Profeta (1877), and Geigel, all opposed to the theory of contagion; Bumstead, Galligo, Belhomme and Martin (1864), Davasse (1865), and Lancereaux and Scarenzio (1866), who are not committed to either side of the question.

One of the latest arguments upon this subject has been presented to us in the form of an experiment made by Voss (1), of St. Petersburgh, the results of which, as they have been widely published, have attracted a great deal of attention. Voss expressed some milk from the breast of a syphilitic female, and, by means of a Pravaz syringe, injected the fluid beneath the integument of three prostitutes. One was syphilitic, and no result followed. The second, affected with urethritis, suffered no ill effects. The third had local swelling, resulting in suppuration at the site of inoculation, but this in a week was relieved. In 40 days, however, a papular eruption appeared near the point where the virus was inserted, and, a few days later, a maculo-papular exanthem spread over the body. The disease was subdued by mercurial treatment.

The result of the first two inoculations being merely negative, we are concerned merely with the conclusion which might be drawn from a superficial examination of the effects of the inoculation in the third case. Was the injected milk the vehicle by which the virus was conveyed to this individual? The only answer which would be justified by the facts given is, that the affirmative has not been unequivocally shown. Obviously a prostitute in hospital does not supply the most favorable field for experimentation of this sort, since the suspicion will arise that she may have suffered from undetected chancre; and may also have been in the incubative period of the disease when submitted to experiment. When we remember what the illustrious Fournier says of the early appearances of chancre in women, declaring in so many words, that it is well nigh impossible not to be mistaken with regard to its character in consequence of its apparent insignificance, we become convinced that only one who is an adept can safely pronounce a prostitute to be free from this disease.

Gallois shows with regard to this case that the extraordinary duration of the incubative period (40 days) is not what might have been expected in view of the fact that in general, after inoculation with the virus of syphilis, this period is shorter than that which succeeds ordinary infection. The history, too, of the initial sore is not that of the typical papule of inoculation. And, lastly, the advent of secondary symptoms in five days after the determination of the primary lesion, is so manifestly opposed to the view that the latter was really the point of entrance of the syphilitic virus into the system, that the case may be discarded so far as it is held to prove the transmissibility of syphilis by the medium of the milk.

It is unnecessary to remark that the somewhat venturesome experimenters of the Italian school, have not failed in the past to test this question by a similar process. Padova inoculated the milk of syphilitic women on six different occasions with negative results, and Profeta, of Palermo, had no better success after as many experiments.

Dismissing, therefore, the questions which are suggested by inoculation with milk supposed to be syphilogenetic, we are confronted with another, of greater interest in a practical point of view, viz., Is there danger for the healthy infant in the ingestion of the milk of a syphiltic nurse? Of course for the syphilitic infant there is no such danger, as it cannot be reinfected with the disease which it has itself already actively and recently manifested. Fournier has called attention to an interesting fact in this connection, that syphilitic nurslings thrive better on the milk of syphilitic nurses than when bottle-fed. Hence, when the nurse has once infected the child at her breast, or the mouth of the child has produced a chancre on the nipple of the nurse, in all cases where it is possible to do so, the two should continue in their former relation for the sake of the child.

In general, of course, there is great danger to be apprehended from placing a healthy infant at the breast of a syphilitic nurse, since the former is almost certain to contract the disease. This result, however, is not due to the infective quality of the nurse's milk, but to the fact that the subjection of the nipple to the gums of the child is liable to irritate the tissues in the former locality, and thus to provoke there the occurrence of mucous patches, or cracks and fissures which may subsequently become transformed into lesions of constitutional syphilis.

The question then is resolved simply into this form: Can the milk of the syphilitic nurse, when not commingled with blood or pathological products, produce the disease in the healthy infant who ingests it?

The clinical facts which authorize a negative answer to this question are numerous, of great interest, and have been furnished largely by the authors whose names have already been given. To these De Amicis (3) contributes an additional observation, and, inasmuch as the history of the case is otherwise instructive in many particulars, it is subjoined in a condensed form:

Annie R., æt. 40, had always enjoyed good health in her youth, and was married in her 22d year. In the first six years after her marriage she had two sons, who are now living and in good health. Her troubles began twelve years ago, when she was nursing her third infant. At that time she had an abundance of breast milk and her child was vigorous, when a neighbor, who had lost her own child, adopted a sickly and emaciated infant taken from a foundlings' home. Sgra. R. was urged to give her breast for a brief time to this sickly child.

The little stranger was nursed for but one day only, as it was then seen to have a sore mouth and an eruption over the skin; and these facts induced Sgra. R. to refuse to nurse it longer. She had reason only too soon to repent of her charitable conduct. A crack appeared upon the left nipple, considerable tumefaction resulted, and she was threatened with suppurative mastitis; but the inflammation subsided under the usual treatment. An induration, however, remained in the site of the fissure, which occasionally pained her. Ignorant of the nature of this disorder, she continued to give her breast to her own healthy child; but, in a few months, she found her own skin covered with a maculo-papular eruption, accompanied by cephalalgia and peri-articular pains. The child, too, soon had ulceration of the buccal cavity, adenopathy, progressive impairment of nutrition and cachexia; it subsequently died.

The mother continued to suffer from pain for several months afterward, and her cutaneous disease rapidly assumed the pustular type (ecthyma). She was submitted to treatment, of the nature of which she is ignorant, including the employment of the thermo-mineral baths of Ischia, without receiving any benefit, the pains increasing and the cutaneous disease remaining unaffected.

Six years elapsed, during which treatment of various character was employed, with no better results, except that she had periods of exacerbation and repose of the malady.

She was seven times pregnant in this interval. The first three pregnancies resulted in abortion between the third and fifth months. In the other four, the children were carried to term, the first two surviving but a few days, the others reaching the age of three years, when they died of typhoid and diphtheritic disorders, without having ever manifested lesions of the skin. After the first two of the last pregnancies—those in which the children died soon after birth—she had again an abundance of breast milk, and dreading its sudden suppression, she suckled two children (one after each pregnancy). These children were then in good health and are now living, vigorous and rosy, their appearance being the best evidence as to their excellent general condition.

In 1874, ten years after her infection, she became pregnant for the last time, and then noticed on her face (especially about the right cheek), the arm of the same side, and the left thigh and foot, certain reddish nodules, which healed after ulceration, leaving more or less deep cicatrices, which are still visible.

This pregnancy was concluded at term, and she bore a well nourished and well developed child, which she nursed, and which remained free from all evidences of disease up to the seventh month. She found, however, that she had not sufficient milk for this infant, and hence arranged with her cousin (whose own child was dead, and whom she believed to be in sound health), that the latter should give the child her own breast. She subsequently discovered that this cousin had a maculo-papular eruption upon the skin, similar to that from which she herself had formerly suffered, as well as rhagades of the nipple. Two months afterward the baby had aphthæ of the mouth, and a skin disease quite like that observed in her own case. The infant was soon relieved of these symptoms; but in 1875 small pustules appeared about the nates and genitals, and spread over the surface from these points. Simultaneously the mother developed tubercles over the right and left elbows, which ulcerated and on one side resulted in the limb being semi-flexed. Other tubercles appeared over the os frontis, sternum and tibia, occasioning excruciating nocturnal pain and more or less distress throughout the year 1876.

Such was the history of the case, and the examination of the patient fully confirmed its essential accuracy. Over the sinciput were various irregularities of surface, due to loss of osseous substance beneath, cicatrices clearly adherent to subjacent tissues, and brownish crusts, beneath which were perforating ulcers of lardaceous aspect. Over the central part of the frontal region, a depressed, adherent, V shaped scar was seen, the osseous tissue centrally having been absorbed, surrounded by peripheral hyperostosis. Over the temporal regions and cheeks were circular and oblong, whitish cicatrices, interspersed here and there with cherry-sized tubercles, some in course of degeneration. Over the manubrium of the sternum was a reddish hemispherical tumor as large as a silver dollar, painful on pressure. Still another depressed adherent cicatrix was found over the left clavicle. An enormous area of cicatricial tissue extended also from the inferior fourth of the right arm to the inferior third of the forearm; indurated, adherent and covered here and there with round, oblong and semi-circular ulcers of foul base. At this point also irregularly shaped cicatricial bridles had produced pseudo-anchylosis of the elbow joint, evidently not due to bicipital contracture. Adherent cicatricial patches, surrounded by clean cut ulcerations, showing a foul base and abundant discharge, extended also from the lower third of the thigh to the inferior fourth of the leg. The diaphysis of the left tibia was double its normal size and tender on pressure. The patient complained of cephalalgia and nocturnal and osteocopic pains. There was no albumen in the urine.

The child, when examined, was found to be emaciated and weak, the head also being disproportioned to the size of the body. Its incisor teeth were not deformed. Yellowish, grey and brown, fatty crusts covered its scalp, beneath which were superficial excoriations of reddish-white aspect and indeterminate margins. There was cervical and submaxillary adenopathy, some of the glands being as large as a pigeon's egg; one, on the right side, had suppurated. Punctiform cicatrices, light reddish brown in hue, were to be seen over the abdomen and back. The tumefied labia majora and minora showed flat, grey, ulcerative patches, which also extended to the anus and nates. The inguino-crural glands were as large as almonds.

The author, of course, does not neglect to draw the conclusions from this case which the facts warrant. The innocuity of the milk of this woman, immediately after each of the pregnancies in which the products of conception, though mature, perished from syphilitic infection, seems to be clearly shown by the immunity and even sound health of her two foster children. Fortunate, indeed, it was for those latter that, during the entire time of lactation, no mucous patch or transformed fissure of the nipple, occurred to menace their safety; for it is well understood to-day, not only that mucous patches may form in the later stages of syphilis, but even that tertiary lesions may furnish infective material, as Bumstead and others have shown.

But De Amicis draws two other conclusions from this clinical history, to which we can accord only partial assent. He formulates the statement that the influence of the syphilitic virus, in itself, does not tend to produce sterility in women, when no change is determined in the organs of generation. This is undoubtedly true, provided it be limited to the fact of conception merely. Practically, syphilis, actively manifested in the female, produces results which are, in the end, not different from those observed in the woman who is sterile; since abortion, due either to placental degeneration or to the blighted ovum itself, is almost pathognomonic of the disease.

Finally, the author concludes, from the fact of the birth of a sound child—the last of the series of pregnancies referred to above — that hereditary syphilis cannot be transmitted in the neoplastic or tertiary stage. This is unquestionably an error, and can readily be refuted by well-established clinical facts. It is true that the power of a parent to transmit syphilis to his offspring wanes with lapse of time; that the sequence of diseased, less diseased, and, finally, healthy children, is a measure of the parental diathesis; and that the chances of sound progeny are greatly increased when treatment has had the effect of time in modifying the disease in the progenitor. But it is also true that the syphilitic father or mother may have diseased children born after they have produced sound infants, and many a father, in the tertiary stage of syphilis, has had occasion to regret the legacy bequeathed to his child.

There is one point upon which De Amicis lays no stress in narrating the history of his case, which merits attention. It is the illustration furnished of the difference between congenital or hereditary syphilis in the infant, and the disease as acquired in infancy. The latter is often named infantile syphilis, though the term is one which misleads many. The two forms of disease are distinctly different. Congenital disease is strictly hereditary. The infant is not poisoned, as was at one time supposed, by an infection occurring at the moment of birth, but is blighted *ab ovo*, so that not merely its cutaneous surface, but its bones and viscera, are liable to display the lesions of the disease. Acquired or infantile syphilis does not differ in its features and career from the acquired disease of the adult, exception being made, possibly, of the tendency of the cutaneous exanthem to assume the form of the mucous patch. This is due to the warmth, moisture and delicacy of the skin of the infant, explained in part by the circumstance that, in very early infancy, the hand is not used in toil, nor the foot in the act of walking.

Although it is not stated that the child from whom Sgra. R. received the disease, was the subject of *congenital* syphilis, still the facts tend to confirm such an opinion. It is described as having gradually become wasted and sickly (*cresceva meschino e malaticcio*), thus suggesting the appearance of premature wrinkled senility, so highly characteristic of the hereditary form. The still-born fœtus and the children dying soon after birth perished of hereditary syphilis. Contrast with these facts the history of the product of the last pregnancy. This child remained well till the seventh month. Few victims of hereditary syphilis survive the fourth month without displaying symptoms of the affection. Arrived at the seventh month, this child acquired the disease as directly as if it had been an adult. Its history, as given by the mother, includes merely a description of adenopathy and cutaneous lesions, confirmed subsequently by a physical examination. Moreover its teeth did not display the changes so minutely described by Hutchinson, as characteristic of the hereditarily syphilitic child.

Dr. Jordan's paper (4) seems to have been prepared with a view to establishing either one of two propositions, and the author does not appear to be greatly interested as to the issue between them. The title of the paper would suggest that the cases are cited in illustration of the theory that the semen of the syphilitic male is *per se* infectious. A similar suggestion is conveyed in the following language: "Syphilis * * * transmitted to the female through the medium of the seminal fluids of the male." Yet in another passage, which refers to the mode of infection of the wives whose cases are reported, the phrase occurs "ladies * * * infected through the medium of the aborted fœtuses." These two propositions differ *in toto*. The one expresses a belief in the infectious character of the semen; the other embodies the doctrine which Diday of Lyons has rather fancifully termed the theory of *choc-en-retour*. In accordance with the latter view, the semen of the syphilitic male transmits hereditary syphilis to the ovum, and this ovum, when it has established intimate relations with the maternal vascular system, poisons the mother. This is really transplacental infection.

The main points in Jordan's case, observed by himself, can be briefly given: A stout and healthy railroad employe had a chancre in 1875, relieved by treatment which, as he states was succeeded by no further lesion. In June of 1876, his wife, 17 years old and seven months pregnant, miscarries with a male child, who utters a moaning, husky cry, has snuffles, and dies in 12 hours. On the 19th of July, the mother has a copper-colored eruption on her face and body, severe iritis and sore throat. She denies having ever suffered from adenopathy or genital sores, and is relieved by anti-syphilitic treatment.

This is a fair example of many reports which are constantly made to medical literature, with a view to establishing one side or the other of a disputed question. For a case to be conclusive on any point, the fullest details and corroborative evidence of every kind are requisite. How much more needful is this when the point is one in controversy! A case fully and conscientiously reported, is an accurate digest of the operations of natural laws, and carries its own corroboration upon its face.

In the instance given above, the sole evidence possessed by the author as to the non-existence of chancre in the wife, is the statement of the latter to that effect! But how few are those married women who know when they suffer from such lesions! Hutchinson has recently declared that it was a useless cruelty to ask a married woman whether she had ever had a chancre, and that her testimony on this point was valueless. Allusion has been already made in these pages to the frequently insignificant character of the primary lesion in women. Even the expert may fail to discern such, in scrutinizing carefully some thirty square inches of vaginal mucous membrane. Had Dr. Jordan, when he wrote his paper for the benefit of the readers of the *American Journal of Obstetrics*, never treated women for non-specific abrasions and ulcerations of the os tincæ, the existence of which they had not dreamed of till his speculum brought them to the light?

By carefully collating the dates given, it will be clear to any one who will take the trouble to investigate the problem, that the chancre of the male, in this case, could scarcely have been healed at the time of his marriage.

But it is not needful to suppose that a syphilitic husband has infected his wife through the medium of the semen, merely because he has no external lesion. It should be remembered that a single drop of his blood is capable of producing the disease in his partner. I have elsewhere* enlarged somewhat upon this point in discussing Diday's recent paper on Syphilis by Conception, and do not therefore dwell further upon it at this time.

The second case reported by Dr. Jordan was observed by Dr. J. C. Mobley, of Winnsboro, S. C., the history of which is said to be given also in the October number of the *Charlestown Medical Journal and Review*. It is briefly this:

A young man, aged 24, examines himself carefully every day for three weeks, after indulgence with a woman of the town, and discovers nothing amiss until a suppurating adonopathy occurs in

---

* "On the Immunity of Certain Mothers of Children Affected with Hereditary Syphilis," a paper read before the American Dermatological Association, Sept. 5, 1877.

the groin. For one year and a half he is under observation, without exhibiting any suspicious symptoms, when he marries, and his wife aborts in the third month. She then exhibits a pigmented syphilide, loses a luxuriant growth of hair, and, in the next year, has a still-born child in the seventh month.

This case also is so loosely reported, that no clue is afforded as to the accuracy of statement or powers of observation of the author. If it bears in any way upon the question of the transmissibility of syphilis in the semen, it will answer equally well as an argument in favor of the saliva, the sweat, or the tears. It is impossible to interpret intelligently any of the mysteries of pathology from such imperfect data, but the facts narrated, suggest the probability that the adenopathy for which the husband was first treated, was associated with a concealed chancroidal ulcer—one for example just beneath the frenum, where I have discovered chancre in patients who affirmed they had never suffered from such lesions. Allowance should always be made for the ignorance of individuals unskilled in observations of this character. For example, I was consulted on the 10th of last November, by an intelligent gentleman of this city, for the relief of an inguinal enlargement, which he declared had always existed, but which he fancied had lately increased in size. I questioned him carefully as to the existence of any venereal sores, and he stated in the most positive manner, that he had never displayed such lesions. I at once proceeded to test the value of this statement by examination, and called his attention to a crust-capped, split-pea sized, indurated nodule, upon the right side of the cutaneous surface of a readily retractable prepuce, in full view of his own eyes as well as of mine. Nothing could exceed his unassumed astonishment at the discovery; and he admitted at once to the exact date of the intercourse by which he was infected.

As to the syphilis acquired by the wife in the case referred to above, it is most readily explained by supposing that at some time before his marriage, on an occasion when he thought it prudent not to consult Dr. Mobley, the husband contracted syphilis, and communicated the disease to his wife in the usual manner.

How often in the history of medicine has a beautiful net-work of speculation and theory been sundered by the lancet of the experimenter! The modest paper of Dr. Hippolyte Mireur (5), solves the problem under consideration in a very simple and straightforward manner, although it must be admitted that, outside of France, an author would scarcely venture to conduct his experiments and afterward to publish the results. Indeed, Mireur admits that a sense of delicacy obliges him to be reticent as to certain details respecting the individuals who have had the boldness to take part in a procedure which many would consider culpable.

A syphilitic patient affected with adenopathy, maculo-papular syphilides, mucous patches of the mouth and impetiginous crusts on the hairy scalp, had never been subjected to specific treatment. He was 26 years of age, and his chancre had cicatrized, leaving a characteristic induration.

The semen of this individual was obtained, and, due care being taken to maintain it at the proper temperature, it was, immediately after its production, used for the purpose of inoculating four persons absolutely free from syphilitic antecedents. All the instruments employed were new and scrupulously clean.

The inoculation of the first two was practiced in the ordinary manner, by means of a grooved needle; six punctures were made in each case—three on each arm. In the third subject, a blister as large as a thimble was produced upon the right leg, the epidermis removed, and a pledget of lint, soaked in the seminal fluid, applied over the derma. This was carefully dressed, and left *in situ* for twenty-four hours.

The fourth was inoculated by the precedure employed by Pellizari in the case of Bargioni, Rossi and Passigli. Near the insertion of the deltoid of the right arm, the epidermis was scraped away, and three small transverse incisions made; the seminal fluid being applied in the manner last described. The wound was dressed, and the whole kept in place for thirty-six hours.

In the instance of the first two individuals, slight local inflammation ensued a few hours after the operation, but this was readily reduced, leaving minute ecchymotic points, which, in five or six days were indiscernible.

In the case of the other two individuals, there was not even this slight degree of reaction. All four were minutely and rigorously inspected daily for ten weeks, and then kept under observation for six months. During this period, none exhibited the slightest evidence of syphilis ; and two of them, examined one year after the inoculation, still remained exempt.

Mireur, anticipating the objections which might be made to definite conclusions from this series of experiments, answers them briefly as follows :

It may be said that the number of individuals inoculated was small. True, he responds ; but if he has not established a certainty, he has, at least raised a presumption of very great probability in favor of the non-contagiousness of the sperm in syphilis.

If it be objected that the semen, exposed to changes by the peculiar conditions of its production and preservation, might have lost its contagious qualities, which it would have retained in the passages of the female, the author would point to the well-known fact of the length of time during which spermatozoids preserve their vitality under much more unfavorable conditions than those obtained in his experiments. It is also well known that the syphilitic virus, when isolated, retains its power to produce the disease for definite periods of time, even when exposed to the air on instruments, transplanted teeth, etc.

But it may be claimed that the virulent principle is localized in the spermatozoa only, and not diffused through the seminal fluid, and that the virus would be so far destructive to the activity and vitality of these elements, as to render the fluid useless as a material for inoculation. To this objection, Mireur replies by pointing to the well-recognized fact that syphilis, though diffused throughout the human organism, is not known to diminish the procreative power of infected males. Besides, he has submitted to microscopical examination the semen of such subjects at various times, and always found the elements perfectly developed and vigorous. If the virus affected these cells to any extent, would it not rather tend to destroy them (as do heat, cold, acids, alkalis, bile, mucus, altered vaginal secretions, etc.,) than to bring about their gradual degeneration and lowered vitality ?

" But," say others, perhaps, " the virus is contained in the spermatic element, and, as the latter cannot enter the circulation, the inoculation gave negative results." It is answered that all the doctrines of contagion teach that mere contact between a virulent germ and a surface deprived of epithelium, is sufficient to ensure this result. Besides, granting that the virus and the spermatic elements are united, it is not necessary to believe that only mature cell elements contain the poison; for the seminal liquid is made up not only of mature elements, but of undeveloped cellules, which, by this hypothesis, would be virulent. These, like blood corpuscles, are susceptible of undergoing absorption, and would thus be capable of transmitting the disease to a healthy individual, had they any such virulent quality.

Drs. Maury and Dulles (6) report a series of cases of acquired syphilis, which are, in many particulars, exceedingly interesting and instructive. In this connection, however, we are concerned merely with the light which they throw upon the questions respecting the saliva.

A vagrant, named James Kelly, contracted four or five chancres, in February, 1877. On the 14th of April, he was treated in the Pennsylvania Hospital for mucous patches of the mouth and condylomata about the anus, and, June 20th, returned with the same disorder. In October, he was detained in the House of Correction.

This man was a "professional" tattooer, and his mode of operation was the following: "Selecting a figure from a book of plates, he would rub up India ink with water, and prick the outlines in with a few needles set in a holder. Then putting the needles in his mouth and sucking out the residue of pigment, he would thrust them, thus moistened, into a bottle of powdered vermilion, and insert what adhered. To renew the vermilion, the needles were repeatedly wetted in his mouth. In some cases, both pigments were moistened with saliva; and in others, he spit upon the finished tatoo, and rubbed it well with his hand, or with a dirty cloth."

For the six months during which this man had lesions of the mouth, he steadily did his work, and the number of his victims—those whom he infected with syphilis by inoculating them with his saliva mixed with the secretion of mucous patches—is not known. Twenty-two of the men exposed to this danger were examined by the authors. Of these, fifteen had unmistakable syphilis, resulting from the tattooing. Three had more or less recently suffered from syphilis before the operation, and, of course, were not reinfected. Four seemed to have escaped entirely.

Was it simply the saliva of a syphilitic man that induced the disease in the fifteen who displayed lesions, or was it such saliva mixed with secretion from the mucous patches in the mouth?

The answer to this is very explicit. The saliva evidently did not produce the result, since it is expressly stated that in each of the four persons who were tattooed, *and who yet escaped infection*, either the *pigments were mixed with Kelly's saliva*, or *when water was used as a substitute*, *the operator repeatedly inserted the needles into his mouth* for the purpose of removing the India ink.

Why did these four escape—one of them tattooed on two different occasions? The conclusion is irresistible that it was not the saliva which was capable of infecting; and that for some reason, by accidental cleansing of the mouth or temporary amelioration of the condition of the buccal cavity, the mucous patches did not, at the time these four men were subjected to the operation, furnish the highly contagious secretion which proved so disastrous to the rest.

Were it not exceeding the scope of this article, it would be interesting to show further that even some of the pathological products in syphilitic individuals are incapable of producing the disease in a healthy person. Diday, upon one occasion, inoculated with the contents of an acne pustule produced by the iodide of potassium in a syphilitic patient, with negative results. And it is now generally known that the contents of the vaccine vesicle in a syphilitic infant, can be introduced with impunity beneath the skin of a healthy child, and be even followed by normal vaccinia; provided always, that no blood nor inflammatory products are introduced at the same time.

I have thus attempted to collate a few recently communicated facts bearing upon the question of the contagiousness of some of the physiological secretions in syphilis; and am persuaded that their careful consideration will warrant the conclusion that we have no evidence to prove that these secretions, when unmingled with blood and pathological products, are capable of transmitting the disease, whether they be introduced by ingestion or inoculation.

